# Acid Ceramidase Inhibition Disrupts Ceramide Homeostasis and Induces Mitochondrial Apoptosis in IDH1-Mutant Oligodendroglioma

**DOI:** 10.21203/rs.3.rs-9077389/v1

**Published:** 2026-03-25

**Authors:** Mioara Larion, Helena Muley, Faris Zaibaq, Meili Zhang, Dionne Davis, Michael Kruhlak, Samarth Mathur, Adrian Lita, Hua Song, Wei Zhang, Zalman Wong, Taylor Harmon, Lumin Zhang, Chen Cam-El Makranz, Aiguo Li, George Karadimov, Fengchao Lang, Christel Herold-Mende, Chunzhang Yang, Tyrone Dowdy

**Affiliations:** Center for Cancer Research (CCR), National Cancer Institute, National Institutes of Health; Center for Cancer Research (CCR), National Cancer Institute, National Institutes of Health; Center for Cancer Research (CCR), National Cancer Institute, National Institutes of Health; Center for Cancer Research (CCR), National Cancer Institute, National Institutes of Health; Center for Cancer Research (CCR), National Cancer Institute, National Institutes of Health; National Cancer Institute; Office of Science and Technology Resources, National Cancer Institute, National Institutes of Health, Bethesda, Maryland, USA; Center for Cancer Research (CCR), National Cancer Institute, National Institutes of Health; Center for Cancer Research (CCR), National Cancer Institute, National Institutes of Health; Center for Cancer Research (CCR), National Cancer Institute, National Institutes of Health; Center for Cancer Research (CCR), National Cancer Institute, National Institutes of Health; Center for Cancer Research (CCR), National Cancer Institute, National Institutes of Health; Center for Cancer Research (CCR), National Cancer Institute, National Institutes of Health; Center for Cancer Research (CCR), National Cancer Institute, National Institutes of Health; Center for Cancer Research (CCR), National Cancer Institute, National Institutes of Health; Center for Cancer Research (CCR), National Cancer Institute, National Institutes of Health; NCI，National Institutes of Health; University of Heidelberg; Center for Cancer Research (CCR), National Cancer Institute, National Institutes of Health; Center for Cancer Research (CCR), National Cancer Institute, National Institutes of Health

**Keywords:** Acid ceramidase, SABRAC, ceramide metabolism, endoplasmic reticulum stress, apoptosis, oligodendroglioma, IDH1 mutation

## Abstract

Oligodendroglioma is genetically defined by mutations in isocitrate dehydrogenase 1 or 2 (IDH1/IDH2) and 1p/19q codeletion. We previously showed that in IDH1-mutant oligodendroglioma, the oncometabolite D-2-hydroxyglutarate biases the sphingosine-1-phosphate–to–ceramide rheostat toward ceramides. Taking advantage of this intrinsic metabolic vulnerability, we investigated whether further elevating ceramide levels through inhibition of acid ceramidase could exacerbate this imbalance and promote apoptotic cell death.

Analysis of patient datasets demonstrated that acid ceramidase is expressed at higher levels in both low- and high-grade gliomas compared with normal tissue. Pharmacologic inhibition of acid ceramidase with SABRAC preferentially reduced viability in human IDH1-mutant oligodendroglioma cell lines. In these sensitive models, acid ceramidase inhibition markedly increased ceramide levels and induced coordinated sphingolipid remodeling. Subcellular imaging using a fluorescent ceramide analogue demonstrated increased ceramide localization to lysosomes and mitochondria following acid ceramidase inhibition. This was accompanied by cytochrome c redistribution, executioner caspase activation, and caspase-dependent apoptotic cell death, consistent with engagement of intrinsic mitochondrial apoptosis. Transcriptomic and biochemical analyses further revealed activation of endoplasmic reticulum stress and unfolded protein response signaling, including PERK- and IRE1α-associated programs, suggesting coordinated multi-organelle stress responses under sustained ceramide elevation. These mechanistic effects translated into a survival benefit in oligodendroglioma xenograft-bearing mice.

Together, these findings suggest that IDH1-mutant oligodendroglioma harbors a pre-existing heightened sensitivity to ceramide stress and identify acid ceramidase as a therapeutically actionable target in this disease.

## Introduction

Oligodendroglioma, although rare, is the third most common glioma in adults (1). An estimated 1,131 people with oligodendroglioma are diagnosed every year in the United States, most often in people between the ages of 35 and 44. Diagnosis of oligodendroglioma is based on the finding of two genetic alterations: mutations in one of the two genes called isocitrate dehydrogenase 1 or 2 (IDH1/IDH2) and the absence of the two chromosomal arms, 1p and 19q (2). Although patients with IDH1-mutant (IDH1^mut^) glioma exhibit a longer survival than those with IDH1 wild-type glioblastoma (GBM), this brain tumor still recurs and may undergo malignant transformation to a higher grade. Additionally, oligodendrogliomas are more prone to develop a hypermutation phenotype which is also associated with a worse prognosis (3). The current therapies for even the highly treatment-responsive oligodendrogliomas are not curative (4,5),highlighting the need to identify tumor-specific vulnerabilities rooted in the unique metabolic features of this disease.

One metabolic pathway that has emerged as a central regulator of cancer cell fate is sphingolipid metabolism. Sphingolipids are a class of bioactive lipids that influence membrane organization, cellular stress responses, and apoptosis (6). A large body of work has focused on the dynamic balance between intracellular ceramide and sphingosine-1-phosphate (S1P) levels, often referred to as the S1P–ceramide rheostat. High ceramides are generally associated with growth arrest and apoptosis, whereas high S1P levels promotes cell proliferation and survival (7–9). In GBM (IDH1-wild-type glioma), dysregulation of the S1P–ceramide rheostat is well established and contributes to multiple malignant hallmarks (10). Consistently, reduced ceramide levels and elevated S1P levels correlate with increasing glioma grade (11). In contrast, the status and functional relevance of this rheostat in IDH1^mut^ glioma were previously unknown. In our prior study, we demonstrate for the first time that accumulation of the oncometabolite D-2-hydroxyglutarate shifts the S1P–ceramide balance toward ceramide accumulation, thereby priming IDH1^mut^ glioma cells for apoptosis. Importantly, we exploited this imbalance by decreasing S1P levels, resulting in preferential cell death in IDH1^mut^ glioma models (Supplementary Fig. S1) (12,13). However, how elevated ceramide levels impact cellular fate in IDH1^mut^ glioma remained unclear.

Herein, we hypothesized that increasing ceramide levels might exploit this metabolic imbalance and promote apoptosis in IDH1^mut^ glioma cells via unique cell death mechanisms compared with decreasing S1P levels. To induce ceramide accumulation, we pharmacologically inhibited acid ceramidase (AC) using SABRAC (N-[(2S,3R)-1,3-dihydroxyoctadecan-2-yl]2-bromoacetamide), a potent and selective irreversible inhibitor. SABRAC is a covalent inhibitor that targets the catalytic cysteine residue (Cys143) of AC, an N-terminal nucleophile hydrolase, and selectively inhibits AC activity without affecting neutral ceramidase or unrelated cysteine proteases (14,15). Using this approach, we delineated the downstream stress signaling pathways and apoptotic mechanisms triggered by ceramide accumulation in oligodendroglioma cells, the most sensitive IDH1^mut^ glioma cells to AC inhibition.

## Results

### *ASAH1* is upregulated in glioma and is associated with oligodendroglioma aggressiveness

**Figure 1:**
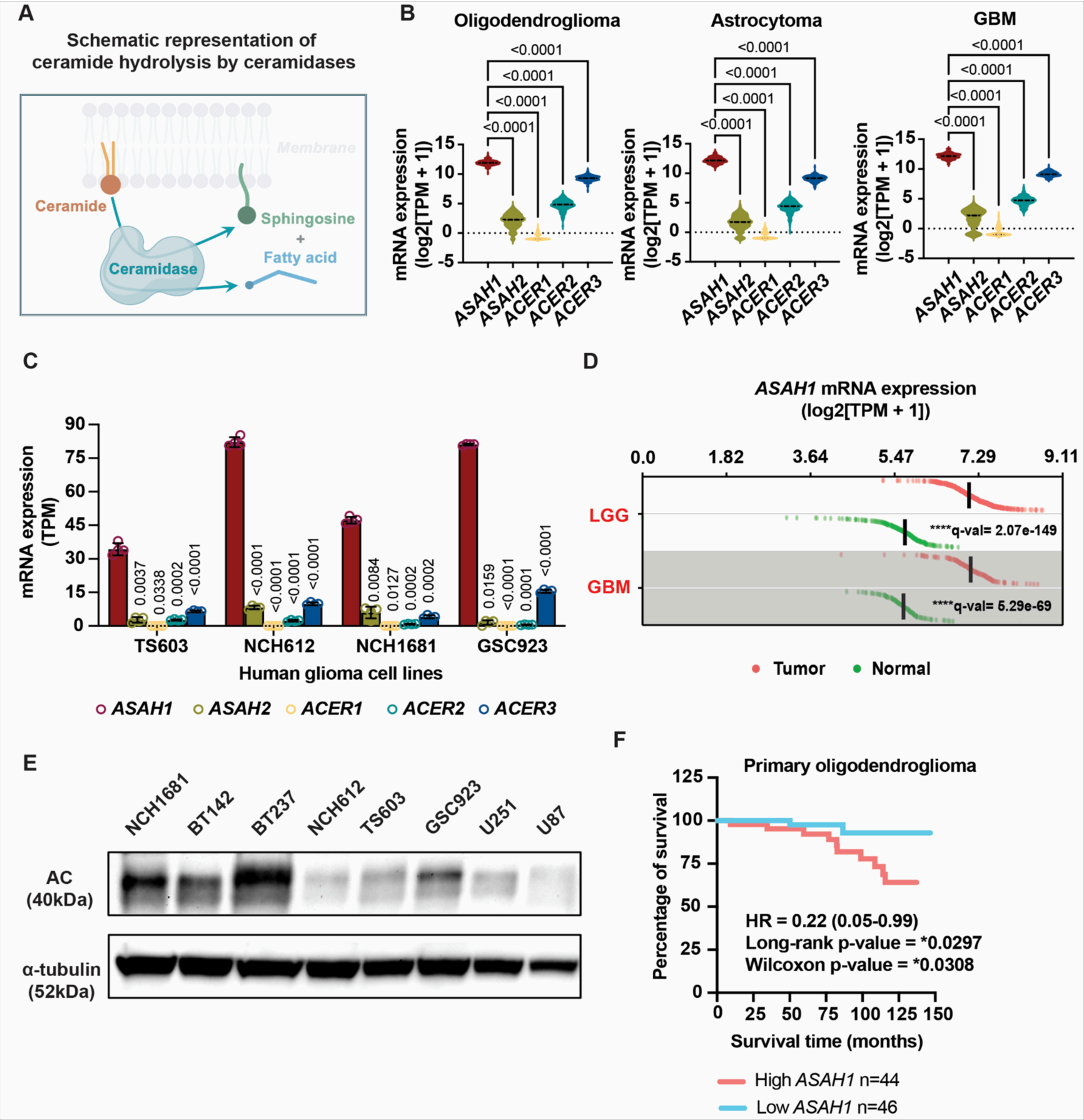
*ASAH1*expression in gliomas. A. Schematic illustration of ceramidase-mediated hydrolysis of ceramide into sphingosine and free fatty acids. B. Violin plots showing log-normalized RNA-seq expression of the five human ceramidases in TCGA oligodendroglioma (n = 191), astrocytoma (n = 194), and glioblastoma (GBM; n = 152) samples. The black horizontal line indicates the median. Within each subtype, matched expression values from the same patients were analyzed using a Friedman test followed by Dunn’s multiple comparisons test (each gene vs *ASAH1*). C. Bar graphs showing TPM expression levels of the five human ceramidases derived from RNA-seq of TS603 and NCH612 (oligodendroglioma), NCH1681 (astrocytoma), and GSC923 (glioblastoma) cell lines (n = 4 biological replicates per cell line). Normalized abundance values were log-transformed and analyzed by two-way repeated-measures ANOVA (including interaction) with Geisser–Greenhouse correction. Comparisons were performed versus the corresponding *ASAH1* condition within each cell line, with FDR control using the Benjamini–Krieger–Yekutieli method; FDR-adjusted p values are shown. Bars represent mean ± SD with individual replicate values shown. D. GEPIA3 expression distribution analysis of *ASAH1*in TCGA low-grade glioma (LGG) (n = 523) and glioblastoma (GBM) (n = 166) compared with GTEx normal brain tissues (LGG comparison: n = 209; GBM comparison: n = 110, including five TCGA paired adjacent normal samples). Each dot represents an individual sample ranked by expression level; vertical bars indicate median expression. Statistical significance was assessed using the limma method implemented in GEPIA3 (**** p < 0.0001). E. Representative immunoblot analysis of acid ceramidase (AC) protein expression in LGG and GBM cell lines. LGG cell lines include NCH1681 (astrocytoma) and BT142, BT237, NCH612, and TS603 (oligodendroglioma). GBM cell lines include GSC923, U251, and U87. a-tubulin served as a loading control. F. Kaplan–Meier survival analysis of primary oligodendroglioma patients stratified by *ASAH1* expression. Overall survival of patients with primary oligodendroglioma from the Chinese Glioma Genome Atlas (CGGA) was analyzed by stratifying cases into high and low expression groups based on tumor expression levels of *ASAH1*. Kaplan–Meier curves are shown, with hazard ratio (HR), log-rank p value, and Wilcoxon p value indicated in the plot.

To identify key regulators of ceramide metabolism in glioma, we first focused on ceramidases, critical enzymes that degrade ceramide and regulate sphingolipid rheostat signaling (16,17). We therefore examined the expression of the five known human ceramidases—*ASAH1*–*2* and *ACER1*–*3*—, which catalyze the hydrolysis of ceramide to sphingosine and free fatty acids (Fig. 1A), across glioma subtypes. Analysis of The Cancer Genome Atlas (TCGA) datasets using the GlioVis data portal (18) revealed that, among ceramidase family members, *ASAH1* (encoding acid ceramidase, AC), exhibited the highest mRNA expression in oligodendroglioma, astrocytoma, and GBM (Fig. 1B). Consistent with these findings, RNA sequencing (RNA-seq) analysis of four patient-derived glioma cell lines representing the three major molecular subtypes (oligodendroglioma: TS603, NCH612; astrocytoma: NCH1681; and GBM: GSC923) demonstrated that *ASAH1* was the most abundantly expressed ceramidase at the transcript level across all cell lines (Fig. 1C). Together, these data indicate that *ASAH1* is broadly enriched across glioma subtypes, prompting us to focus subsequent analyses on this enzyme. To further define the clinical relevance of *ASAH1* expression, we compared its mRNA levels in glioma with those to normal brain tissue. Using GEPIA3 platform (19), we found that *ASAH1* mRNA levels were significantly higher in both low-grade glioma (LGG) and GBM compared with normal brain samples (Fig. 1D and Supplementary Table S1). Consistent with transcriptomic data, *ASAH1* protein expression was detected in LGG- and GBM-derived human glioma cell lines by immunoblotting (Fig. 1E and Supplementary Fig. S2).

We next assessed the association between *ASAH1* expression and patient outcome. Kaplan–Meier survival analysis of patient data from the Chinese Glioma Genome Atlas (CGGA) indicated an association between *ASAH1* mRNA expression and patient outcome in IDH1^mut^ oligodendroglioma (Fig. 1F). No significant survival correlation was observed for *ASAH1* mRNA expression in CGGA astrocytoma or GBM cohorts, nor in TCGA-based analyses across the major glioma subtypes, including oligodendroglioma, astrocytoma, and GBM, (Supplementary Fig. S3). Overall, survival analyses indicated that associations between *ASAH1* mRNA expression and outcome were cohort- and subtype-specific, while *ASAH1* mRNA expression was consistently elevated in glioma relative to normal brain tissue.

### Pharmacological inhibition of AC reduces glioma cell viability and promotes ceramide accumulation with mitochondrial enrichment

**Figure 2:**
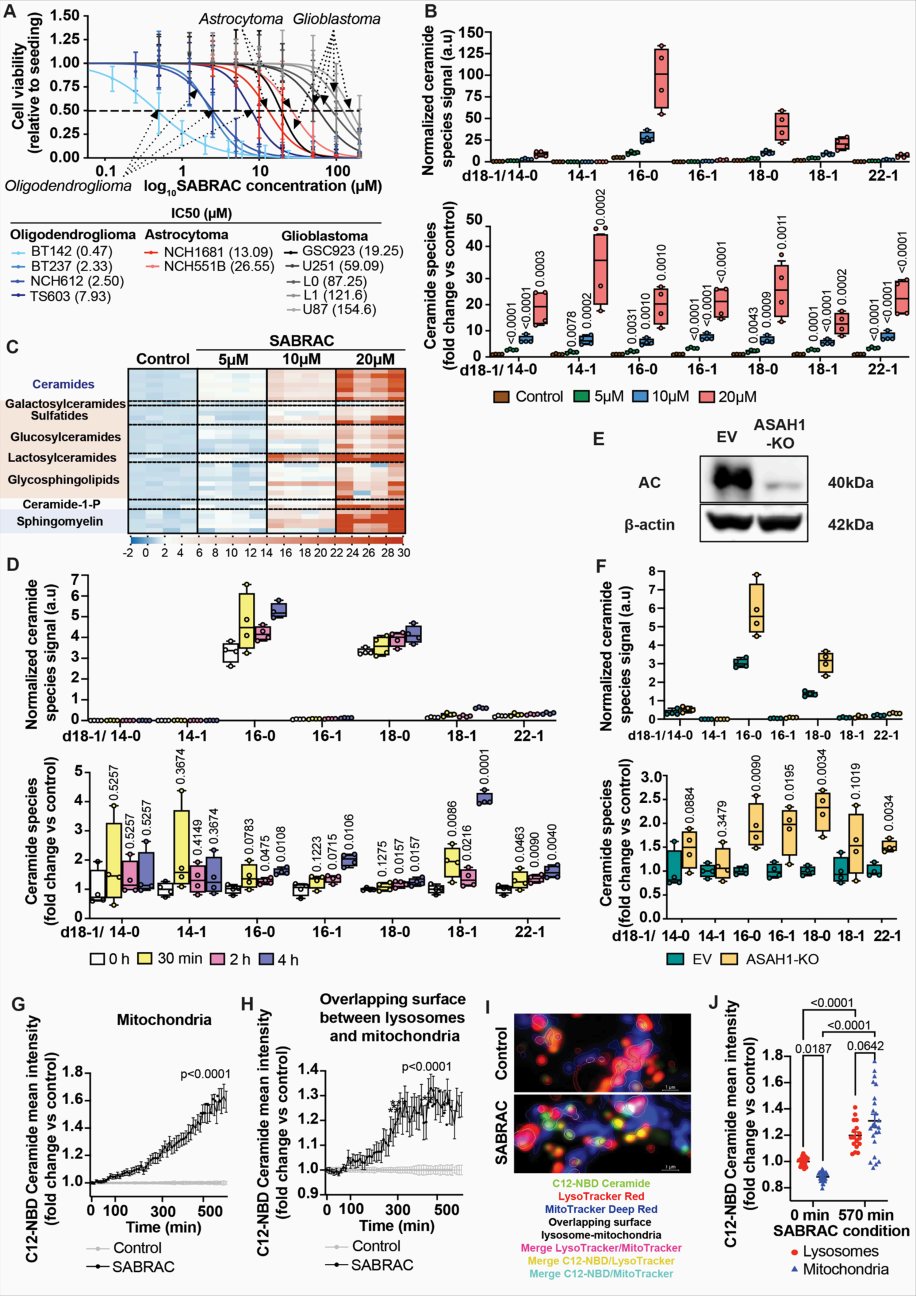
Effects of SABRAC on glioma cell viability and sphingolipid metabolism. A. Dose-response analysis. Cell viability was assessed using the Cell Counting Kit-8 assay following 48 h exposure to SABRAC (0–200 μM) in human glioma cell lines. Values were normalized to the mean of untreated controls (0 μM), set to 1. Data represent at least three biological independent experiments, each performed with ≥3 technical replicates per concentration. Dose-response curves were fitted using nonlinear regression (four-parameter logistic, concentration vs response) by ordinary least squares, with the top plateau constrained to 1 and the bottom plateau constrained to 0. IC_50_ was defined as the concentration corresponding to 50% viability. IC_50_ values for each cell line are shown in the figure, and 95% confidence intervals, Hill slope, R^2^, Sy.x, and degrees of freedom are provided in Supplementary Table S2. All curves shown represent converged fits. B, C, D, F. Quantification of sphingolipids in oligodendroglioma cells by LC–MS. For box-plot panels, ceramide levels are shown as abundance normalized to internal standards and protein content. Individual points represent biologically independent replicates (n = 4 per group). Boxes indicate the interquartile range with median; whiskers denote minimum-maximum values. Top panels show normalized ceramide species abundance. Bottom panels represent the identical dataset, expressed as fold change relative to control. (B) Ceramide abundance in NCH612 cells treated with 0, 5, 10, or 20 μM SABRAC for 48 h. (C) Heatmap from the same experiment as in (B) showing fold change relative to control in ceramides and other sphingolipids. (D) Ceramide abundance in NCH612 cells treated with 10 μM SABRAC for the indicated times (0 h, 30 min, 2 h, 4 h). (F) Ceramide abundance in ASAH1-KO and empty vector (EV) NCH612 cells. For panels B, D, and F, normalized abundance values were log-transformed and analyzed by two-way repeated-measures ANOVA (including interaction) with Geisser–Greenhouse correction. Comparisons were performed versus the corresponding control condition within each ceramide species, with FDR control using the Benjamini–Krieger–Yekutieli method; FDR-adjusted p values are shown. E. Representative immunoblot images confirming ASAH1-KO with β-actin as loading control. G–J. Confocal microscopy of fluorescent C12-NBD ceramide showing subcellular distribution in control and SABRAC-treated cells, including lysosomes, mitochondria, and mitochondria–lysosome overlapping surface. Data are representative of three independent biological experiments; each performed with eight technical replicates. (G, H) Graphs showing how SABRAC increases C12-NBD Ceramide in mitochondria and the overlapping surface between lysosomes and mitochondria in TS603 cells. Ceramide fluorescence intensities were normalized by time 0 and the control values. Results are shown as mean ± SEM. Regression analysis followed by comparison of slopes was applied (n= 24 fields—5 z-stacks and 57 time points for each one—per condition). (I) Confocal images from Control- and SABRAC-treated cells showing the regions of interest (ROI, lysosomes, mitochondria and overlapping surface between lysosomes and mitochondria) that were automatically selected for C12-NBD Ceramide fluorescence quantification. Ceramide increase under SABRAC treatment is highlighted by the presence of merged colors as yellow (ceramide in lysosomes) and cyan (ceramide in the mitochondria). (J) Graph showing C12-NBD Ceramide in lysosomes and mitochondria over time. Two-way ANOVA test was applied (n= 24 fields—including 5 z-stacks for each one—per condition).

Given the overexpression of AC at the transcript level in glioma tissues and cell lines (Fig. 1), we next investigated whether pharmacological inhibition of AC exerts antitumor effects. Treatment with the AC inhibitor SABRAC (14,15)(Supplementary Fig. S4), significantly reduced cell viability, with IDH1^mut^ oligodendroglioma cell lines displaying greater sensitivity compared with other glioma subtypes, followed by IDH1^mut^ astrocytoma and finally IDH1-wild-type GBM (Fig. 2A, Supplementary Table S2 and Fig. S5). Additional structurally related irreversible AC inhibitors (SOBRAC, SOCLAC, and SACLAC), were also evaluated in BT142 IDH1^mut^ oligodendroglioma cells (Supplementary Fig. S6). Based on its lower IC_50_, SABRAC was selected for subsequent mechanistic studies.

To further characterize the metabolic consequences of AC inhibition, we performed LC–MS-based lipidomic profiling of two patient-derived IDH1^mut^ oligodendroglioma (NCH612 and TS603) cell lines treated with increasing concentrations of SABRAC (5–20 μM). Consistent with its catalytic function, SABRAC-mediated AC inhibition induced robust ceramide accumulation in NCH612 IDH1^mut^ oligodendroglioma cell line, as quantified by LC–MS-based lipidomics. This increase occurred across multiple ceramide species in a dose-dependent manner (Fig. 2B) and was similarly observed in an independent IDH1^mut^ oligodendroglioma cell line (TS603) (Supplementary Fig. S7). Interestingly, LC–MS profiling also revealed a concomitant increase in ceramide-derived downstream sphingolipids following SABRAC treatment (Fig. 2C and Supplementary Fig. S8 and S9). In line with these lipidomic changes, RNA-seq performed following SABRAC treatment in both NCH612 and TS603 IDH1^mut^ oligodendroglioma cell lines revealed coordinated upregulation of genes involved in glycosphingolipid metabolism, consistent with ceramide-driven metabolic rewiring (Supplementary Fig. S10 and S11).

Although SABRAC was originally developed and biochemically characterized as a selective covalent inhibitor of AC (14,15), we sought to determine whether the accumulation of ceramides and their derivatives induced by SABRAC treatment occurs specifically through inhibition of this enzyme. To address this, we first analyzed the short-term effects of SABRAC by performing a time-course experiment in the IDH1^mut^ oligodendroglioma cell lines NCH612 and TS603, treating cells with 10 μM SABRAC for 0.5, 2, and 4 hours, followed by LC–MS-based lipidomic analysis. In NCH612 cells, SABRAC induced a progressive accumulation of multiple ceramide species. Cer 18:1 and Cer 22:1 increased significantly as early as 30 min and remained elevated at 2 and 4 h. Cer 16:0 and Cer 18:0 showed significant increases at 2 and 4 h, whereas Cer 16:1 increased significantly only at 4 h. In contrast, Cer 14:0 and Cer 14:1 remained unchanged in these short time points (Fig. 2D). In TS603 cells, only one of the eight detected ceramide species showed a statistically significant increase at 4 h, whereas two additional species demonstrated a near-significant upward trend (Supplementary Fig. S12). To further examine AC’s contribution to these elevated ceramide levels, we generated genetic ASAH1 knockout (ASAH1-KO) models in both cell lines. In NCH612 cells, ASAH1-KO resulted in significant increases in Cer 16:0, Cer 18:0, Cer 16:1, and Cer 22:1 (Fig. 2E and 2F). Notably, Cer 16:0 and Cer 18:0—the most abundant ceramide species in these cells—were among those significantly elevated and together comprise the dominant fraction of the total ceramide pool. In contrast, ASAH1-KO in TS603 cells did not result in statistically significant changes in any ceramide species (Supplementary Fig. S13). In NCH612 cells, ASAH1-KO partially recapitulated the ceramide profile observed after short-term SABRAC treatment, supporting the interpretation that early ceramide accumulation occurs, at least in part, through on-target AC inhibition. In contrast, neither ASAH1 ablation nor short-term SABRAC exposure produced robust ceramide increases in TS603 cells. However, prolonged SABRAC treatment induced a marked increase in all ceramide species in both cell lines, indicating that additional mechanisms likely contribute to the extensive ceramide accumulation observed at later time points.

To further validate SABRAC’s inhibitory activity at the subcellular level, we monitored ceramide distribution using the fluorescent analogue C12-NBD ceramide and live-cell confocal microscopy (Fig. Supplementary Fig. S14). Because AC localizes to lysosomes, we first quantified lysosomal ceramide accumulation, which progressively increased following SABRAC exposure and became significant at 260 min, reaching a plateau by 570 min (Supplementary Fig. S14). In parallel, mitochondrial C12-NBD signal increased with slightly earlier kinetics, becoming significant at 240 min and continuing to rise throughout the observation period without clear saturation (Fig. 2G). In addition, ceramide C12-NBD signal progressively increased within regions of overlapping fluorescence between lysosomes and mitochondria (Fig. 2H and 2I). Notably, whereas baseline C12-NBD ceramide levels were lower in mitochondria than in lysosomes, SABRAC treatment led to progressive mitochondrial enrichment such that the levels of C12-NBD ceramide were reversed by 570 min (Fig. 2J). Together with lipidomic analyses demonstrating early accumulation of total ceramides—particularly C16:0, a well-established pro-apoptotic species, and C18:0, which has been linked to apoptotic signaling in select contexts (20–24) as early as 2 h—these spatial and temporal dynamics are consistent with rapid ceramide buildup in lysosomes followed by redistribution/enrichment toward mitochondria after AC inhibition.

### SABRAC treatment induces mitochondrial-dependent apoptosis in oligodendroglioma cells

**Figure 3:**
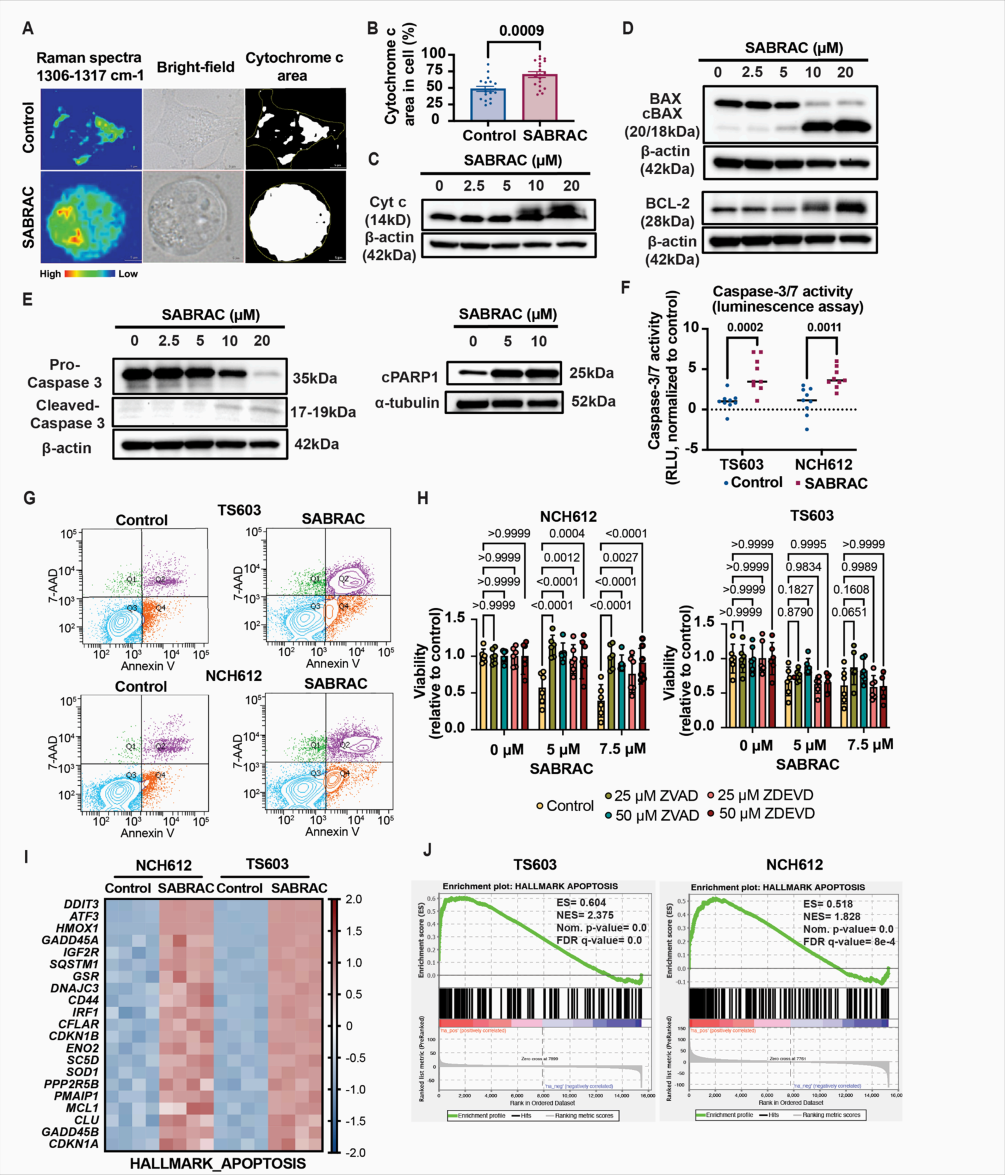
SABRAC treatment induces intrinsic apoptotic signaling in oligodendroglioma cells. TS603 and NCH612 oligodendroglioma cell lines were treated with SABRAC at the indicated concentrations for 48 h unless otherwise specified. A–B. Raman analysis demonstrating increased cytosolic cytochrome c (Cyt c) in TS603 cells following 5 h treatment with 10 μM SABRAC compared with vehicle control. Raman spectral profiles were fixed at 1306 and 1317 cm^−1^. A. Representative Raman images, brightfield images, and corresponding ImageJ analyses are shown. B. Quantification of cytosolic Cyt c signal expressed as percentage of Cyt c-positive area per cell. Five independent biological experiments were performed, each with at least four technical replicates per condition. Eighteen control cells and nineteen SABRAC-treated cells were analyzed. Individual values are shown with mean ± SEM. Statistical significance was determined using an unpaired two-tailed Student’s t-test after confirmation of normal distribution (Shapiro–Wilk test). C. Immunoblot analysis confirming increased Cyt c protein levels following SABRAC treatment. D. Immunoblot analysis of intrinsic apoptotic regulators in TS603 cells treated with increasing concentrations of SABRAC (2.5–20 μM), showing modulation of BAX, cleaved BAX, and BCL2. E. Immunoblot analysis of apoptotic execution markers demonstrating decreased pro-caspase-3 and increased cleaved caspase-3 and cleaved PARP1 following treatment. Immunoblots (C–E) were repeated in at least two independent biological experiments with comparable results; representative blots are shown. β-actin served as a loading control. F. Caspase-3/7 activity measured by luminescent assay in TS603 and NCH612 cells treated with 10 μM SABRAC for 24 h. Statistical analysis was performed using two-way ANOVA followed by Sidak’s multiple comparisons test. Three independent biological experiments were performed in triplicate. Individual values are shown, with the mean indicated by a horizontal bar. G. Flow cytometric analysis of apoptosis using Annexin V and 7-AAD staining in TS603 and NCH612 cells treated with 7.5 μM SABRAC for 16 h. Experiments were conducted in at least two independent biological replicates per cell line. H. Cell viability rescue assessed by Cell Counting Kit-8 assay following co-treatment with SABRAC (5 or 7.5 μM) and either the pan-caspase inhibitor zVAD-FMK or the caspase-3/7 inhibitor zDEVD-FMK. Statistical analysis was performed using two-way ANOVA followed by Dunnett’s multiple comparisons test. Data represent mean ± SD with individual values shown. At least two independent biological experiments were performed, each in triplicate. I. Heatmap of Hallmark apoptosis leading-edge gene expression in NCH612 and TS603 cells following SABRAC treatment. TS603 and NCH612 oligodendroglioma cell lines were treated with 7.5 μM SABRAC. Owing to differential sensitivity to the drug, NCH612 cells were treated for 24 h and TS603 cells for 6 h. RNA-seq was performed on n = 4 independent biological replicates per group. Differential expression analysis was performed using DESeq2. Expression values were row-scaled using transformed expression data. Leading-edge genes were defined by preranked GSEA of the Hallmark apoptosis gene set to highlight transcriptional responses shared across both cell lines. J. GSEA demonstrating enrichment of the Hallmark apoptosis gene set in both cell lines. Enrichment score (ES), normalized enrichment score (NES), nominal p-value, and false discovery rate (FDR q-value) are indicated in the graph.

Given the established role of mitochondrial ceramide accumulation in promoting mitochondrial outer membrane permeabilization (25–28), we next examined whether SABRAC treatment activates the intrinsic apoptotic pathway in oligodendroglioma cells by assessing cytochrome c release. Raman imaging revealed increased cytosolic localization of cytochrome c in SABRAC-treated cells (Fig. 3A, 3B and Supplementary Fig. S15 and Fig. S16), consistent with mitochondrial outer membrane permeabilization and occurring after the early ceramide redistribution observed in Fig. 2G–J. Increased cytochrome c levels were further confirmed by immunoblot analysis (Fig. 3C). Analysis of mitochondrial apoptotic regulators demonstrated dose-dependent modulation of BCL-2 family proteins, including increased cleaved BAX and altered BCL-2 expression in IDH1^mut^ oligodendroglioma TS603 (Fig. 3D) and NCH612 (Supplementary Fig. S17) cell lines following SABRAC treatment. Consistent with engagement of apoptotic execution pathways, SABRAC treatment resulted in decreased pro-caspase-3 levels and increased cleavage of caspase-3, PARP1 and caspase-8 (Fig. 3E and Supplementary Fig. S18). Increased caspase-3/7 activity was further confirmed by luminescence assay in both TS603 and NCH612 cells (Fig. 3F). Flow cytometric analysis using Annexin V and 7-AAD staining confirmed increased apoptotic cell populations following SABRAC treatment in both cell lines (Figure 3G). Importantly, pharmacologic inhibition of caspases rescued SABRAC-induced loss of viability in NCH612 cells, while TS603 cells showed a more limited response under the conditions tested (Fig. 3H), supporting a caspase-dependent mechanism of cell death. At the transcriptional level, RNA-seq analysis of NCH612 and TS603 IDH1^mut^ oligodendroglioma cells treated with SABRAC, revealed consistent regulation of apoptosis-related genes across both cell lines (Fig. 3I), and Gene Set Enrichment Analysis (GSEA) confirmed significant enrichment of the Hallmark Apoptosis gene set (Fig. 3J). Both classic and pre-ranked GSEA approaches yielded highly concordant enrichment profiles across all comparisons; therefore, enrichment plots and associated statistics shown throughout the manuscript are derived from the pre-ranked analysis.

Collectively, these findings demonstrate that AC inhibition induces mitochondrial outer membrane permeabilization followed by caspase-dependent apoptosis in IDH1^mut^ oligodendroglioma cells, consistent with a model in which ceramide accumulation drives mitochondrial apoptotic signaling.

### Ceramide accumulation activates ER stress and the unfolded protein response in oligodendroglioma cells

In addition to promoting mitochondrial apoptotic signaling, ceramide accumulation has been well established to disrupt endoplasmic reticulum (ER) homeostasis (29–31). We therefore investigated whether SABRAC-induced ceramide elevation (Fig. 2B, 2D and 2F) engages ER stress and unfolded protein response (UPR) signaling in oligodendroglioma cells. Using the RNA-seq dataset described above, we next examined activation of ER stress-related pathways.

**Figure 4:**
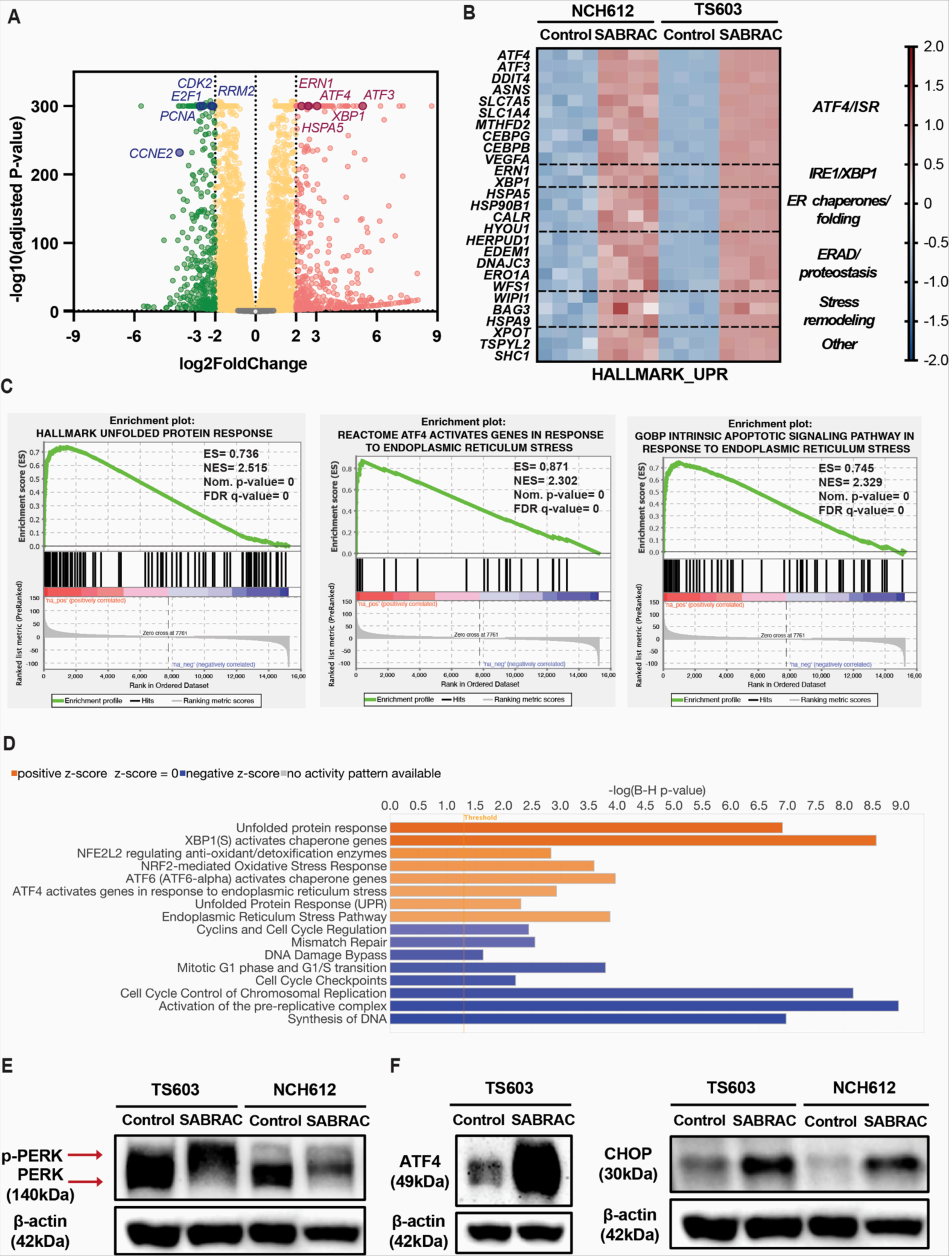
SABRAC induces ER stress and activates the unfolded protein response (UPR) in IDH1^mut^oligodendroglioma cells. Cells were treated with 7.5 μM SABRAC for 24 h (NCH612) or 6 h (TS603). A–D. Transcriptomic analysis of SABRAC-treated cells by RNA-seq. RNA-seq was performed on n = 4 independent biological replicates per group. A. Volcano plot depicting differential gene expression in NCH612 cells following SABRAC treatment, generated from DESeq2 analysis. Selected ER stress and UPR-associated genes (ATF4, ATF3, ERN1, XBP1, HSPA5) and representative DNA replication and cell-cycle regulators (CDK2, E2F1, PCNA, CCNE2, RRM2) are highlighted. B. Heatmap of Hallmark unfolded protein response (UPR) leading-edge gene expression in TS603 and NCH612 cells following SABRAC treatment. Expression values were row-scaled using transformed expression data. Leading-edge genes were defined by preranked GSEA of the Hallmark UPR gene set to highlight transcriptional responses shared across both cell lines. Genes are organized by functional category, including ATF4/ISR-associated targets, IRE1/XBP1 components, ER chaperone/folding genes, ER-associated degradation and proteostasis machinery, stress remodeling, and other stress-associated genes. C. GSEA performed in NCH612 cells following SABRAC treatment demonstrating significant enrichment of ER stress- and apoptosis-related transcriptional programs, including the Hallmark UPR gene set, Reactome ATF4-dependent signaling in response to ER stress, and GO Biological Process intrinsic apoptotic signaling pathway in response to ER stress. Enrichment plots are shown for representative gene sets. Enrichment score (ES), normalized enrichment score (NES), nominal p-value, and false discovery rate (FDR q-value) are indicated in the graph. D. Ingenuity Pathway Analysis of differentially expressed genes in NCH612 SABRAC-treated cells highlighting activation of ER stress-associated signaling pathways, including UPR, XBP1-mediated chaperone activation, ATF4-associated stress responses, and NRF2/NFE2L2-regulated oxidative stress pathways, alongside suppression of pathways related to cell-cycle progression and DNA replication. Bars represent -log10(Benjamini–Hochberg adjusted p-values) for pathways with predicted activation or inhibition. E. Immunoblot analysis of ER stress signaling demonstrating increased phosphorylation of PERK in SABRAC-treated cells compared with control conditions. PERK activation is indicated by the upward mobility shift of the phospho-PERK band. β-actin was used as a loading control. F. Immunoblot analysis in TS603 and NCH612 cells showing ATF4 and CHOP protein expression following SABRAC treatment, with β-actin as a loading control.

Differential expression analysis in NCH612 cells revealed a pronounced transcriptional shift characterized by activation of ER stress pathways and suppression of proliferative programs. Volcano plot analysis demonstrated significant upregulation of canonical UPR and ER stress-associated gene transcripts, including *ATF4*, *ATF3*, *ERN1*, *XBP1*, and *HSPA5*, alongside downregulation of key regulators of DNA replication and cell-cycle progression, such as *CDK2*, *E2F1*, *PCNA*, *CCNE2*, and *RRM2* (Fig. 4A). Similar transcriptional trends were observed in SABRAC-treated TS603 cells (Supplementary Fig. S19) indicating a consistent stress-responsive shift across models. To determine whether these changes reflected coordinated activation of ER stress programs, we examined leading-edge genes driving enrichment of the Hallmark UPR gene set. Heatmap analysis of significantly enriched leading-edge genes identified by pre-ranked GSEA and shared between TS603 and NCH612, demonstrated concordant upregulation following SABRAC treatment (Fig. 4B). Functional categorization revealed coordinated activation of multiple UPR branches, including ATF4-dependent targets of the integrated stress response (ISR), IRE1–XBP1 pathway components, ER chaperones and protein-folding genes, as well as ER-associated degradation (ERAD) and other proteostasis regulators, together with additional stress-remodeling genes. These data demonstrate activation of a conserved, multi-branch ER stress transcriptional program in response to SABRAC in both oligodendroglioma cell lines. GSEA further substantiated activation of ER stress signaling. In both NCH612 (Fig. 4C) and TS603 (Supplementary Fig. S20) cell lines, significant enrichment was observed for the Hallmark UPR, Reactome ATF4-dependent signaling in response to ER stress, and the Gene Ontology (GO) Biological Process intrinsic apoptotic signaling pathway in response to ER stress gene sets (Supplementary Tables S3–10).

To integrate these transcriptional changes at the pathway level, Ingenuity Pathway Analysis (IPA) was performed in NCH612 cells using significantly regulated genes. IPA identified robust activation of ER stress-associated pathways, including UPR signaling, XBP1-mediated activation of chaperone genes, ATF4-associated stress responses, and NRF2/NFE2L2-regulated antioxidant and detoxification pathways. In addition, IPA revealed significant suppression of pathways related to cell-cycle progression and DNA replication, including cyclin signaling, G1/S transition, cell-cycle checkpoints, activation of the pre-replicative complex, and DNA synthesis pathways (Fig. 4D). Although IPA also detected ATF6-associated gene expression signatures, direct activation of ATF6 was not assessed in this study (Supplementary Fig. S21, S22 and Tables S11, S12).

To validate activation of ER stress signaling at the protein level, we next examined key mediators of the UPR. Immunoblot analysis revealed increased phosphorylation of PERK following SABRAC treatment in both TS603 and NCH612 cell lines, evidenced by an upward mobility shift of the PERK band relative to control conditions (Fig. 4E). Consistent with activation of the PERK arm of the UPR, SABRAC treatment resulted in marked induction of ATF4 and the pro-apoptotic transcription factor CHOP in both cell lines (Fig. 4F and Supplementary Fig. 23). In addition, the ER stress-associated protein p8 was increased following treatment (Supplementary Fig. S24), further supporting activation of ER stress signaling.

Collectively, these findings demonstrate that SABRAC-induced ceramide accumulation is associated with a conserved, multi-branch ER stress program prominently engaging the PERK-ATF4 axis, while simultaneously suppressing proliferative pathways. Together with mitochondrial apoptotic activation, these data support a model in which ceramide accumulation coordinates organelle stress responses culminating in apoptotic cell death (Fig. 6).

### Pharmacologic AC inhibition prolongs survival in an oligodendroglioma xenograft model

**Figure 5:**
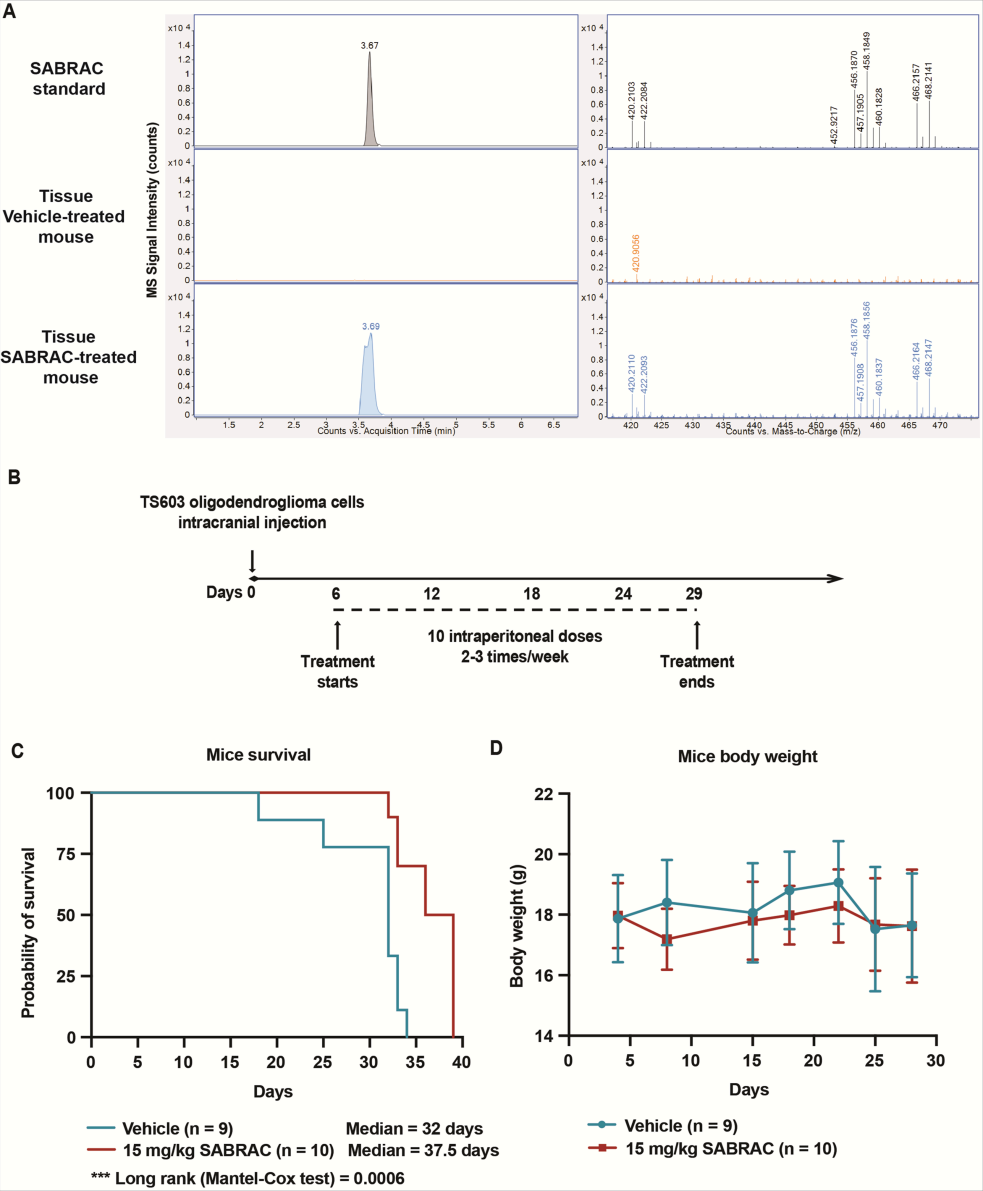
SABRAC treatment confers a survival benefit in an orthotopic oligodendroglioma xenograft model. A. LC–MS-based detection of SABRAC in brain tissue. Brain samples were obtained from mice included in a SABRAC toxicity study. Brains were collected 4 h after intraperitoneal (i.p.) administration of SABRAC (15 mg/kg) or vehicle. Left panel: Extracted ion chromatograms of [SABRAC+Cl^−^]^−^ at m/z 458.186. Right panel: Extracted mass spectra within the corresponding peak retention time window, showing the characteristic bromine isotope pattern of SABRAC. The ions [SABRAC-H^+^]^−^, [SABRAC+Cl^−^]^−^, and [SABRAC+CHO_2_^−^]^−^ were detected at m/z 420.2104, 456.1870, and 466.2158, respectively. SABRAC detection was confirmed in two independent brain samples from mice treated at 15 mg/kg. B–D. In vivo efficacy and tolerability of SABRAC in an orthotopic oligodendroglioma xenograft model. Female 8-week-old SCID mice were intracranially injected with TS603 human oligodendroglioma cells to establish orthotopic tumors and subsequently randomized to receive vehicle (n = 9) or SABRAC (15 mg/kg, i.p., n = 10) according to the dosing schedule shown in (B). B. Schematic representation of the treatment regimen. C. Kaplan–Meier survival analysis. Animals were monitored daily and euthanized upon reaching predefined humane endpoints. Survival was analyzed using the log-rank (Mantel–Cox) test; median survival and exact p value are indicated in the plot. D. Body weight (grams) was measured longitudinally in the same cohort of mice and is presented as mean ± SD. Mixed-effects model (RMEL) analysis showed an effect of time (p = 0.0068), but no effect of treatment (p = 0.554) or treatment × time interaction (p = 0.0948). Šídák’s multiple comparisons test detected no differences between groups at any time point.

Having established that SABRAC induces ceramide accumulation, mitochondrial apoptosis, and ER stress signaling *in vitro*, we next asked whether pharmacologic inhibition of AC confers therapeutic benefit *in vivo*. We therefore evaluated SABRAC tolerability and brain exposure in SCID mice prior to efficacy studies. Animals received intraperitoneal injections of SABRAC (1, 5, or 15 mg/kg) or vehicle five times per week. SABRAC was well tolerated at all tested doses, with no significant body weight loss or overt signs of toxicity observed during treatment (Supplementary Fig. 25). To assess systemic drug exposure and brain penetration, plasma and brain tissues were collected 4 h after the final administration and analyzed by LC–MS. SABRAC was detectable in brain tissue at 15 mg/kg, confirming central nervous system penetration at this dose (Fig. 5A). Given that 15 mg/kg represented the highest well-tolerated dose, it was selected for therapeutic evaluation in the oligodendroglioma xenograft model. Mice bearing orthotopic oligodendroglioma xenografts treated with SABRAC exhibited a significant extension of survival compared with vehicle-treated controls, as assessed by Kaplan–Meier analysis (median survival 37.5 days vs. 32 days; log-rank Mantel–Cox test, p = 0.0006) (Fig. 5B and 5C). Throughout the treatment period, mice maintained stable body weight, with no significant differences observed between groups (Fig. 5D), further supporting tolerability of the regimen. Collectively, these data indicate that SABRAC reaches the brain following systemic administration at therapeutically relevant doses and confers a significant survival benefit in an oligodendroglioma xenograft model, supporting its therapeutic potential *in vivo*.

## Discussion

Metabolic determinants of cell death susceptibility remain incompletely defined in molecularly stratified gliomas. Although alterations in sphingolipid metabolism are increasingly recognized as determinants of cancer cell fate (6,32), how these pathways can be therapeutically leveraged across distinct glioma subtypes is still unclear. Building on our prior observation that IDH1^mut^ gliomas exhibit a ceramide-biased sphingolipid rheostat relative to IDH1-wild-type tumors (12,13), the present study identifies AC as a subtype-relevant metabolic vulnerability in IDH1^mut^ oligodendroglioma. Our findings suggest that disrupting ceramide catabolism shifts lipid homeostasis beyond a tolerable threshold, triggering coordinated organelle stress responses centered on mitochondrial apoptotic activation and ER stress signaling. Importantly, this mechanistic vulnerability translates into measurable survival benefit in an orthotopic intracranial model, supporting AC inhibition as a rational therapeutic strategy in this disease context.

Comparative transcriptomic analyses across glioma cell lines and patient cohorts demonstrated that *ASAH1* is the predominant ceramidase expressed in glioma, with substantially higher expression than other ceramidase family members and marked elevation relative to non-neoplastic brain tissue. This expression hierarchy suggests that *ASAH1* represents a major contributor to ceramide turnover in glioma and reinforces its functional relevance as a regulator of sphingolipid homeostasis in these tumors. While *ASAH1* expression was broadly elevated across glioma grades relative to non-neoplastic brain tissue, its prognostic association appeared most pronounced within oligodendroglioma. Variability across datasets and subtypes is not unexpected given differences in cohort composition, molecular stratification, and treatment heterogeneity (33). Importantly, the functional sensitivity observed in oligodendroglioma models is consistent with this subtype-enriched survival association, reinforcing the biological relevance of AC in this context.

Although both astrocytoma and oligodendroglioma are IDH1-mutant, oligodendroglioma patient-derived cell lines appeared more sensitive to AC inhibition with SABRAC, as reflected by lower IC_50_ values. One possible explanation for this differential vulnerability lies in additional metabolic consequences of 1p/19q codeletion, a defining feature of oligodendroglioma. Co-deleted IDH1^mut^ gliomas exhibit significantly reduced expression of PHGDH and CTH, enzymes involved in serine biosynthesis and transsulfuration pathways that support glutathione production and redox buffering (34), suggesting a constrained ability to manage oxidative and organelle stress. In this context, ceramide accumulation induced by AC inhibition may more readily overwhelm ER and mitochondrial stress-adaptive mechanisms in oligodendroglioma compared with astrocytoma, lowering the threshold for intrinsic apoptotic commitment.

Prolonged pharmacologic AC inhibition with SABRAC resulted in robust accumulation of multiple ceramide species in both oligodendroglioma models at 48 h (Fig. 2), indicating effective disruption of ceramide catabolism. In addition to accumulation of long-chain ceramides, SABRAC treatment was accompanied by coordinated increases in complex sphingolipids, including glucosylceramides, lactosylceramides and sphingomyelins. This pattern is consistent with activation of downstream metabolic buffering pathways that attempt to redistribute excess ceramide rather than isolated accumulation of a single lipid species. Despite this compensatory remodeling, sustained elevation of pro-apoptotic long-chain ceramides persisted, suggesting that buffering capacity is insufficient to prevent mitochondrial apoptotic activation under conditions of AC inhibition.

In NCH612 IDH1^mut^ oligodendroglioma cells, early time-course and AC genetic ablation experiments further demonstrated significant increases in the predominant long-chain ceramides C16:0 and C18:0, together with several additional species. In contrast, TS603 IDH1^mut^ oligodendroglioma cells exhibited more modest early kinetic responses: genetic ablation did not produce statistically significant changes, and short-term SABRAC exposure resulted in significant elevation of only a minor species (C14:0), although C16:0 and C18:1 displayed clear upward trends approaching statistical significance. Despite these quantitative differences in early dynamics between these two patient-derived cell lines, prolonged inhibition converged on marked accumulation of long-chain ceramides. Importantly, C16:0 ceramide has been consistently implicated in BAX activation, mitochondrial outer membrane permeabilization, and intrinsic apoptosis, whereas the pro-death role of C18 ceramide appears more context-dependent (20,21,28).

The dominant elevation of C16:0, together with accumulation of C18:0 under sustained stress conditions, provides a mechanistic basis for the robust mitochondrial apoptotic activation observed in oligodendroglioma cells. Early cytochrome c redistribution and subsequent caspase activation indicate engagement of mitochondrial outer membrane permeabilization as a central death mechanism following SABRAC treatment. Despite quantitative differences in early ceramide kinetics between TS603 and NCH612 cells, both models converged on mitochondrial apoptotic commitment, underscoring a common ceramide-dependent intrinsic death program. The more robust caspase-dependent rescue observed in NCH612 compared with TS603 likely reflects quantitative differences in death pathway engagement rather than distinct underlying mechanisms.

**Figure 6:**
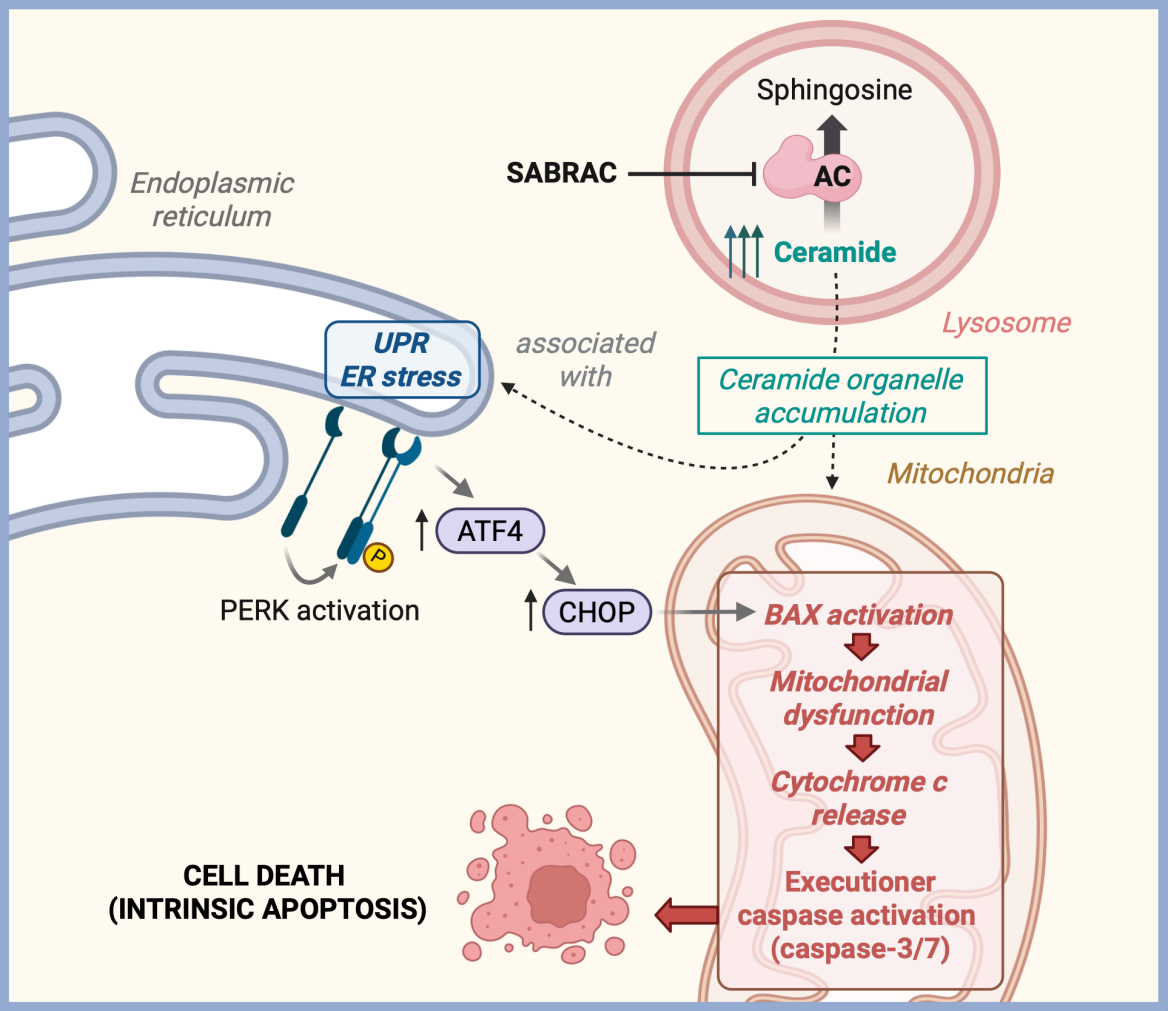
Schematic representation of the proposed mechanism by which SABRAC induces cell death in IDH1^mut^ oligodendroglioma cells. SABRAC, a covalent and irreversible inhibitor of lysosomal acid ceramidase (AC), blocks the hydrolysis of ceramide into sphingosine and free fatty acids, resulting in ceramide accumulation within lysosomes. Ceramide buildup is associated with its redistribution to mitochondria and activation of endoplasmic reticulum (ER) stress and the unfolded protein response (UPR), characterized by PERK phosphorylation and increased ATF4 and CHOP expression. These events promote mitochondrial dysfunction, BAX activation, cytochrome c release, caspase activation, and ultimately intrinsic apoptotic cell death.

Beyond their direct effects on mitochondrial outer membrane permeabilization, ceramides are also known to perturb ER homeostasis. Elevation of ceramide levels has been shown to alter ER membrane properties and disrupt ER Ca^2+^ handling—potentially through modulation of SERCA activity—thereby activating UPR signaling pathways that can transition from adaptive to pro-apoptotic outputs under sustained stress (29,30,35). The coordinated activation of ER stress pathways alongside mitochondrial apoptosis suggests that SABRAC-derived sustained ceramide accumulation engages multi-organelle stress responses that converge on intrinsic cell death. Previous studies in cancer cells have documented ceramide-induced ER stress or mitochondrial apoptosis, but we demonstrate that both pathways are concomitantly engaged in IDH1^mut^ oligodendroglioma (Fig 6.).

Having established this mechanistic framework *in vitro*, we next examined whether AC inhibition translates into therapeutic benefit *in vivo*. SABRAC was readily detectable in brain tissue by LC–MS, demonstrating central nervous system exposure. In an orthotopic intracranial oligodendroglioma xenograft model, SABRAC significantly prolonged survival without inducing measurable weight loss, indicating tolerability at therapeutically effective doses. Although the survival benefit was modest, it is notable given the aggressive nature of intracranial glioma models and the use of single-agent therapy, and it provides proof-of-concept that targeting ceramide catabolism can impact tumor progression *in vivo*.

Collectively, our findings identify AC as a metabolic vulnerability in oligodendroglioma and delineate a mechanistic cascade whereby inhibition of ceramide degradation amplifies ceramide stress, disrupts ER and mitochondrial homeostasis, and activates apoptotic cell death programs. These results highlight the therapeutic potential of exploiting sphingolipid metabolic dependencies in genetically defined glioma subtypes. Future studies aimed at optimizing AC inhibitor pharmacokinetics, and exploring rational combination strategies may further enhance the clinical relevance of this approach. The implications of this study are that targeting AC represents a novel therapeutic strategy for IDH1^mut^ oligodendroglioma, a cancer subtype with limited treatment options at this stage.

## Methods

### GEPIA3 expression analysis in tumor and normal tissues

Publicly available RNA-seq data from TCGA and the Genotype-Tissue Expression (GTEx) project were analyzed using the GEPIA3 web server (19). *ASAH1* expression profiles were generated for TCGA glioma cohorts (LGG and GBM) and compared with GTEx normal brain tissues using the GEPIA3 “Expression DIY” module. When the limma option was selected, GEPIA3 used log2(TPM+1)-transformed TPM values for visualization and statistical testing. Sample sizes for each comparison are reported in the corresponding figure legend.

### TCGA and CGGA glioma expression and survival analyses

Gene expression and survival data from adult glioma patients were obtained from TGCA (TCGA-GBMLGG) and the CGGA. Data were accessed and analyzed using the GlioVis data portal (18). Survival analyses were performed based on stratification by *ASAH1* expression, as described in the corresponding figure legends.

### Cell models

The following human glioma cells were used in this project. GSC923 (GBM) were developed by the Neuro-Oncology Branch (National Cancer Institute). TS603 (oligodendroglioma) were kindly provided by Dr. Timothy Chan’s lab; NCH612 (oligodendroglioma), NCH551B (astrocytoma) and NCH1681 (astrocytoma) by Dr. Christel Herold-Mende’s lab; and L0 (GBM) and L1 (GBM) by Dr. Jinkyu Jung. BT237 (oligodendroglioma) was received from Oligo Nation. BT142 (oligoastrocytoma), U87 MG cells (GBM) and 293T were purchased from ATCC and U251 MG (GBM) from Sigma. U251 MG cells, U87 MG and 293T cells were cultured in DMEM (10–013-CV Corning) supplemented with 10% fetal bovine serum (SH12450H R&D Systems) and 1% penicillin-streptomycin (15140122 Gibco).

### Neurospheres culture

All the experiments were performed using human-derived stem-like spheroids. Neurospheres were grown in suspension in DMEM/F12 medium (11320033 Gibco) supplemented with 1% N2 growth supplement (17502048 Gibco), 2 μg/mL heparin sodium salt (07980 Stem Cell), 20 ng/mL EGF (236-EG R&D Systems), 20 ng/mL FGF (3718-FB R&D Systems) and 1% penicillin-streptomycin (15140122 Gibco).

### Generation of ASAH1-KO Cells

To establish the ASAH1-KO cell line, two AC guide RNA sequences cloned into pLenti-CRISPR v2 plasmid were provided by the NCI LASP Genome Modification Core. The sgRNA sites targeting the *ASAH1* gene were: CGCGACGGCACAGCTGACGGCGG and AAGGCGACGCAACTCCGGCCCGG. The gRNA plasmids were packaged into lentivirus particles using lipofectamine 3000 (Thermo Fisher) in 293T cells (Thermo Fisher) with psPAX2 (Addgene #12260) and pMD2.G (Addgene #12259). The lentiviruses were concentrated with Lenti-X concentrator (Takara) and applied to TS603 and NCH612 oligodendroglioma cell lines. Cells were selected under 1 μg/mL puromycin. The genetic KO of AC was tested by immunoblotting. Both sgRNAs demonstrated similar KO efficiency.

### Cell viability assays

For cell viability assays, cells were plated using optimal seeding densities per well in 96-well plates with neurosphere growth medium and drug treatments. Cell viability was assessed by Cell Counting Kit-8 (CK04 Dojindo). The absorbance was measured at 450 nm. The concentration of the drug resulting in 50% inhibition of cell viability (IC_50_) was calculated using non-linear regression curve fitting.

### Immunoblotting

Cell lysates were prepared using NP-40 lysis buffer (J60766.AP Thermo Scientific) containing protease inhibitors (A32955 Thermo Scientific). The lysates were centrifuged at 13,000 × g for 10 min to obtain the supernatants. BCA assays (23225 Thermo Scientific) were performed for measuring protein concentration. The proteins in the lysates were resolved by SDS-polyacrylamide gel electrophoresis and were transferred to LF PVDF membranes (Bio-Rad). The immobilized proteins were immunoblotted with antibodies against the proteins of interest. The bands were visualized using an enhanced chemiluminescence (ECL) system (ChemiDoc MP BIO-RAD). Equal loading of proteins was verified by immunoblotting for β-actin or a-tubulin. All the antibodies used in immunoblot analysis are listed below. Rabbit anti-AC (HPA005468 Sigma-Aldrich), mouse anti-IDH1 (R132H) (SAB4200548 Sigma-Aldrich), rabbit anti-PERK (3192S Cell Signaling), rabbit anti-ATF4 (11815S Cell Signaling), rabbit anti-Caspase 8 (13423–1-AP Proteintech), rabbit anti-BCL-2 (12789–1-AP Proteintech), rabbit anti-BAX (5023 Cell Signaling), rabbit anti-Cytochrome c (11940T Cell Signaling), rabbit anti-Caspase 3 (19677–1-AP Proteintech), rabbit anti-cleaved PARP1 (ab32064 abcam), rabbit anti-phospho-BAD (9291S Cell Signaling), rabbit anti-BAD (ab32445 abcam), rabbit anti-p8 c-terminal (SAB2109172 Sigma), rabbit anti-phospho-eIF2α (9721S Cell Signaling), rabbit anti-eIF2α (9722S Cell Signaling), rabbit anti-CHOP (15204–1-AP Proteintech), rabbit anti-TRIB3 (13300–1-AP Proteintech), mouse anti-beta actin (ab6276 abcam), mouse anti-beta actin (66009–1-Ig Proteintech), mouse anti-α-Tubulin (DM1A) (3873S Cell Signaling), peroxidase-conjugated AffiniPure goat anti-rabbit IgG (111–035-144 Jackson) and peroxidase affiniPure goat anti-mouse IgG (115–035-146 Jackson).

### Liquid chromatography-mass spectrometry (LC–MS)-based lipidomics

#### Sample preparation

TS603 and NCH612 oligodendroglioma cells (5 × 10^6^ cells per condition) were treated with SABRAC at the concentrations and time points indicated in each figure. DMSO-treated cells served as controls. In parallel, empty vector (EV) and ASAH1-KO cells were collected without SABRAC treatment. Cells were collected by centrifugation at 300 × g for 5 min and washed with phosphate-buffered saline (PBS). All remaining PBS was removed using an additional centrifugation step. Cell pellets were snap-frozen on dry ice and stored at −80 °C until LC–MS analysis. Mouse brain tissues were collected, snap-frozen on dry ice, and stored at −80 °C until analysis.

#### Chemicals and Reagents

All solvents used were LC–MS or better quality, except for HPLC-grade ethanol-stabilized chloroform (CHCl3). Acetonitrile (ACN), 2-propanol (IPA), and water were procured from Honeywell Scientific. Formic acid (FA) and ammonium formate (AF) were purchased from Fisher Scientific (Waltham, MA, USA). Methanol (MeOH) and CHCl3 were bought from GFS Chemicals.

#### Sphingolipid and polar lipid optimized extraction

Cell sample extraction was performed as described in Dowdy T. et al, 2020 (13). The only modification from the protocol was related to the solvent composition used for reconstituting the hydrophilic and hydrophobic phases that was 100 μL of 5:4:1 EtOH/MeOH/water.

Brain tissue was homogenized via sonication probe for 20 s (~30 J). Suspension was centrifuged at 10,000 × g for 5 min at 4 °C, and supernatant was collected. All samples were normalized to 2.5 mg/mL protein in water per BCA assay prior to modified Folch extraction. All extraction steps were performed on wet ice. Briefly, 1 mL MeOH was added to 250 μL diluted homogenate, then 2 mL CHCl_3_. Solution was shaken 30 min at 4 °C. Layer separation was induced via addition of 500 μL water. Samples were shaken 10 min at 4 °C and centrifuged at 310 rcf for 5 min at 4 °C. Bottom (organic) layer was collected. Samples were reextracted with 1 mL 2:1 CHCl_3_:MeOH, shaken 10 min at 4 °C, and centrifuged at 310 × g for 5 min at 4 °C. Bottom (organic) layer was collected and combined with that from previous step. Extract was dried under N_2_. Sample was reconstituted first in 100 μL 2:1 CHCl_3_:MeOH and allowed to incubate at 4 °C for 15 min before addition of 100 μL 1:1 IPA:MeOH and 50 μL water.

#### UHPLC–HRMS Methodology

For cell samples, LC/MS analysis were previously described in Dowdy T. et al, 2020 (13), except for the mass accuracy and retention time values. Targeted ion selection, alignment, and annotation for logical binning of the input data were restricted to ion mass accuracy ± 2.0 mDa and retention time ± 0.5 min using an in-house Personal Compound Data Library (PCDL) of known sphingolipids. Following preprocessing, the ion area for each analyte was adjusted to ratio by sample-specific internal standard area and then corrected to sample-specific weight quantification.

For brain samples, UHPLC–HRMS methodology was adapted from Ulmer et al (36). Mobile phases A and B were 10 mM AF, pH 3.5, in 3:2 ACN:water and 10 mM AF, pH 3.5, in 45:4:1 IPA:ACN:water, respectively. Column was Agilent Poroshell C18, 1.9 μm, 2.1 × 100 mm. Needle washes 1, 2, and 3 were 1:1 MeOH:water, 1:1 ACN:water, and 4:3:2:1 IPA:MeOH:ACN:water, respectively. Flow rate was 400 μL/min for separation and 700 μL/min for reequilibration, and column temperature was held at 55 °C. Injection volume was 2 μL for positive mode and 4 μL for negative mode. Run time was 20 min. Gradient elution was as follows: ramp from 25 % to 95 % B over 13 min, flush at 95 % B for 2 min, reset to 25 % B over 0.1 min, hold at 400 μL at 25 % B over 0.9 min, increase flow to 700 μL/min at 25 % B over 0.1 min and hold for 3 min, and decrease flow to 400 μL at 25 % B over 0.1 min. UHPLC–HRMS analyses were performed on an Agilent Infinity II UHPLC coupled to an Agilent 6545 Q-ToF mass spectrometer with dual Jet Stream source. Real-time mass correction was used. Iterative exclusion MS/MS was utilized to identify compounds in pooled QC samples. One reconstitution blank and one extracted blank injection were made to exclude method and extraction artifacts, and six pooled QC injections were used to generate MS/MS spectra for library comparison.

#### Software

Data processing and analysis were performed with MZMine 3.9.0 and MSConvert. Software parameters, batch files, and libraries can be found in supplemental information.

### RNA Sequencing and Bioinformatic Analysis

#### RNA extraction

TS603 and NCH612 oligodendroglioma cells (2.5 × 10^6^ cells per condition) were treated with DMSO or 7.5 μM SABRAC for 6 h and 24 h, respectively. Cells were collected by centrifugation at 300 × g for 5 min, washed once with PBS, and pelleted again. Cell pellets were snap-frozen in dry ice and stored at −80 °C until RNA extraction. Total RNA was purified using the PureLink^™^ RNA Mini Kit (12183025, Invitrogen), including an on-column DNase digestion step using the PureLink^™^ DNase Set (12185010, Invitrogen).

#### RNA Sequencing

RNA integrity was assessed using a 5300 Fragment Analyzer System (Agilent Technologies). RNA Integrity Number (RIN) values were >9.2 for all samples except two (RIN 8.8 and 7.9). As the sequencing facility requires a minimum RIN of 8 for library preparation, all samples met quality control criteria and were submitted to the CCR Sequencing Facility. Paired-end sequencing (2 × 100 bp) was performed on a *NovaSeq* 6000 instrument using the *NovaSeq* 6000 S1 Reagent Kit.

#### Data preprocessing, filtering, and quality control

Sequencing reads of RNA-seq raw data were analyzed using CCBR pipeline RENEE (37) v.2.7.2 (Rna sEquencing aNalysis pipElinE) (https://github.com/CCBR/RENEE). The pipeline performs several tasks: pre-alignment reads quality control, grooming of sequencing reads, alignment to human reference genome (hg38), post-alignment reads quality control, and feature quantification. In the QC phase, the sequencing quality of each sample is independently assessed using FastQC, Preseq, Picard tools, RSeQC, SAMtools and QualiMap. Sequencing reads were trimmed for adaptor and low-quality sequences using Cutadapt algorithm. The trimmed reads were aligned using STAR and then gene and isoform expression was quantified using RSEM. Sample quality control was vigorously carried out based on sequencing criteria such as mapping rates, library complexities, etc. Further sample filtering was performed based on zero value of specific genes and the sample variance.

Gene-level raw count matrices (RSEM gene counts) were assembled for all samples (n = 16; two cell lines TS603 and NCH612 with n = 4 SABRAC-treated and n = 4 Vehicle-treated per cell line). Low-abundance genes were filtered prior to downstream analyses using a counts-per-million (CPM) approach implemented in edgeR, retaining genes with CPM > 0.5 in at least 4 samples (the minimum group size). This filtering strategy removes consistently uninformative low-count features while maintaining an analysis set driven by biologically meaningful expression and adequate power for dispersion/variance estimation, consistent with established RNA-seq filtering recommendations (38). After filtering, 16,766 genes were retained for differential expression and enrichment analyses.

#### Differential Gene Expression Analysis

Differential gene expression analysis was performed using DESeq2 v1.50.2 (39) but separately for each patient-derived glioma cell line (TS603 and NCH612) to estimate treatment effects within each biological background and avoid confounding by baseline transcriptional differences between cell lines. For each cell line, a *DESeqDataSet* object was constructed from filtered count matrices with treatment condition (SABRAC vs. Control) as the design variable. Variance-stabilizing transformation (VST) was applied to normalized counts for visualization and input to GSEA, providing log2-scale data with stabilized variance across the expression range. DESeq2 analysis was executed using default parameters, which employs empirical Bayes shrinkage for dispersion estimation and fits negative binomial generalized linear models to test for differential expression. For each gene, the Wald test statistic was calculated as the ratio of the log2 fold change to its standard error (Wald = log2FC / lfcSE), providing a variance-normalized effect size that accounts for estimation uncertainty—a critical consideration given our sample size of n=4 per group (39). To obtain more stable and interpretable effect sizes for visualization (e.g., volcano plots), log2 fold changes were additionally shrunken using adaptive shrinkage via *ashr* package, which reduces exaggerated estimates for low-information genes while largely preserving ranking among well-supported effects (40). Genes were considered differentially expressed at an adjusted p-value (FDR) threshold of 0.05 using the Benjamini–Hochberg correction for multiple testing.

#### Gene Set Enrichment Analysis

Gene Set Enrichment Analysis (GSEA) was conducted using two complementary approaches to ensure robust pathway-level interpretation: classic matrix-based GSEA and pre-ranked GSEA. Both analyses utilized the MSigDB Hallmark gene set collection (v2025.1), comprising 50 well-defined biological processes derived from multiple curated pathway databases (41).

#### Classic Matrix-Based GSEA

To assess pathway-level responses to SABRAC treatment, we performed matrix-based GSEA separately for each cell line using the original GSEA algorithm framework (42). For each cell line, the VST-transformed expression matrix (genes × 8 samples) was exported with gene identifiers and corresponding descriptions, and a phenotype label file defined the two classes (SABRAC vs Control; n = 4 per class). Gene ranking was performed internally using the Signal-to-Noise ratio metric, which accounts for both effect size and within-group variance—a critical consideration for small sample sizes (43). Enrichment significance was assessed using gene-set permutations (1,000 permutations) as sample-label (phenotype) permutation is not well powered or stable for very small group sizes (n<7; GSEA manual). Genes were ranked internally using the Signal-to-Noise metric, which incorporates both effect size and within-group variability and is the default ranking strategy in the Broad GSEA implementation (42). Enrichment scoring used the weighted statistic (p = 1), and gene set sizes were constrained to 15–500 genes to avoid unstable very small sets while excluding excessively broad sets. Where Ensembl IDs were used in the expression matrix, features were collapsed/remapped to gene symbols using the appropriate MSigDB chip annotation to ensure identifier compatibility with Hallmark symbol gene sets.

#### Pre-ranked GSEA

In parallel, we performed a pre-ranked GSEA analysis to couple enrichment testing directly to the DESeq2 model-based differential expression results and to provide an analysis path fully consistent with the RNA-seq statistical framework (GSEA documentation). For each cell line, all genes with finite DESeq2 Wald statistics were ranked by the signed Wald statistic, which represents a variance-normalized, directional measure of differential expression (positive values indicating higher expression in SABRAC vs Control; negative values indicating higher expression in Control). This ranking metric has been explicitly recommended for DESeq2-based RNA-seq analyses because it encodes effect size, direction, and statistical uncertainty in a single value—precisely what GSEA ranking requires (44). Ensembl version suffixes were removed prior to export, and where multiple entries mapped to the same identifier, the entry with the maximum absolute statistic was retained to avoid duplicate ranking artifacts. Pre-ranked enrichment was performed for 16,711 genes against the same Hallmark gene sets (41) using 1,000 gene-set permutations and with identical parameters (weighted enrichment statistic, 15–500 gene size range, FDR (False discovery rate) q-value < 0.25) yielding normalized enrichment scores (NES) and multiple-testing-adjusted significance estimates.

We implemented both GSEA approaches to validate pathway discoveries across methods, as recommended by recent comparative studies (44). Classic GSEA with Signal-to-Noise ranking provides variance-aware gene ranking computed directly from expression data, while pre-ranked GSEA with Wald statistics leverages the full DESeq2 statistical model, including dispersion shrinkage and outlier detection. Importantly, given our sample size constraint necessitating gene set permutation—which does not account for gene-gene correlation structure in biological pathways—we recognize that statistical significance alone may not reflect meaningful biological distinction (44). Therefore, we prioritized pathways showing (1) FDR q-values < 0.05, (2) |NES| > 1.5, and (3) consistent discovery across both GSEA implementations for biological interpretation and validation experiments. The top 10 pathways are nearly identical between methods, demonstrating that core biological findings are robust to ranking metric choice (Wald statistic vs Signal-to-Noise). Both GSEA implementations identified highly concordant pathway signatures in SABRAC-treated NCH612 cells, with 100% overlap in the top 12 pathways. The dominant biological response—activation of ER stress/unfolded protein response (NES=2.52–2.55) and NF-κB-mediated inflammatory signaling (NES=2.32–2.68)—was robust across both analytical approaches. Minor methodological differences were observed: Basic GSEA with Signal-to-Noise ranking produced stronger enrichment scores for interferon response pathways (NES=2.09 vs 1.75), while pre-ranked GSEA with Wald statistics emphasized cholesterol homeostasis and protein secretion pathways. One pathway (KRAS_SIGNALING_UP) showed methodological sensitivity (FDR=0.001 vs 0.32), but core biological conclusions regarding ER stress, inflammatory signaling, and metabolic reprogramming were invariant to ranking metric choice.

#### Ingenuity Pathway Analysis (IPA)

The complete DESeq2 results table, including log2 fold changes and adjusted p values, was uploaded into Ingenuity Pathway Analysis (IPA, QIAGEN). Within IPA, genes were filtered using an adjusted false discovery rate (FDR) < 0.05; and an absolute log2 fold change (|log2FC|) ≥ 1.0 for NCH612 cells and ≥ 0.75 for TS603 cells. Core analysis was performed using default parameters to identify enriched canonical pathways and predicted upstream transcriptional regulators. Statistical significance was determined using IPA’s right-tailed Fisher’s exact test, and activation states were inferred using activation z-scores. Pathway diagrams and upstream regulator networks were generated using IPA.

#### Confocal microscopy

In a laminin-precoated 8-well coverglass chamber, TS603 cells were seeded in neurosphere media containing 6μM C12-NBD Ceramide. After 5 hours, ceramide was removed and replaced with 10μM SABRAC (treated condition) or DMSO (control condition) and the organelle dyes. After a 3 h-incubation, confocal z-stacks were acquired every 10min over a 7-hour time period, using a Nikon SoRa spinning disk microscope equipped with a 60x Apo TIRF oil immersion objective lens (N.A. 1.49) and Hamamatsu ORCA Fusion BT sCMOS camera. The microscope was equipped with a Tokai Hit stage-top incubation chamber to maintain cells at humidity, temperature (37 °C) and 5% CO_2_. Confocal z-stack images were acquired with 0.110 μm x-y pixel size and 0.45 μm z-step size from multiple positions within each of the wells in the coverglass chamber using the Nikon Elements image acquisition software (v. 5.4.1). Imaris image analysis software (v. 10.1) was used to segment the lysosomes and mitochondria independently as isosurface objects using an intensity-based threshold algorithm. The mean fluorescence intensity of C12-NBD Ceramide was measured over time in the individual segmented lysosomes and mitochondria, and in the overlap regions between lysosomes and mitochondria. Outliers were identified using the ROUT test (Q = 1%) and excluded prior to analysis. C12-NBD Ceramide was acquired from Cayman and the organelle’s dyes (LysoTracker^™^ DND-99 and MitoTracker^™^ Deep Red) were ThermoFisher products.

#### Raman spectroscopy

TS603 cells were grown in serum containing media in 35 mm dishes and were treated with SABRAC at 10μM or with vehicle for 5h. Raman spectra from cells were obtained with a DXRxi Raman confocal microscope (Thermo Scientific) using a 562nm excitation laser, 100X immersed objective, 10mW of laser power, and 1μm slit aperture for 2s/pixel/scan integration time. Spectra were collected using the Thermo Scientific OMNIC Software, integrated, and background corrected. OMNICxi profile Chemigram function was fixed between 1,306 and 1317 cm^−1^ and used for map visualization of Cytochrome c distribution.

#### Fluorogenic caspase 3/ 7 activation assay

Oligodendroglioma cells were grown in a black 96-well plate with transparent round bottom and were treated with SABRAC (10μM) or vehicle for 24h. CellEvent^™^ Caspase-3/7 Red (Invitrogen) fluorogenic reagent (substrate for activated caspase-3/7) was added to the cells. The reagent was prepared as recommended in the datasheet. After 3 hours of incubation, fluorescence was measured at 584/625 wavelengths in a Multi-Detection Microplate Reader Synergy^™^ HT (Bio-Rad, Hercules, CA, USA).

#### Flow cytometry-based apoptosis assay

Oligodendroglioma cells were treated with 7.5 μM drug for 16 h prior to analysis. Apoptosis was assessed by flow cytometry using a FITC-Annexin V/7-AAD staining kit (BioLegend 640922). Briefly, 10^6^ cells per sample were collected, washed once with PBS, and resuspended in 100 μL Annexin V Binding Buffer. Cells were stained with 5 μL FITC-Annexin V and 5 μL 7-AAD and incubated for 15 min. Samples were then diluted with PBS (final volume 1mL) and analyzed within 30 min of staining on a BD FACSCanto II flow cytometer. Debris was excluded by forward- and side-scatter gating, and doublets were removed using FSC-A versus FSC-H gating. Apoptotic populations were defined as viable (Annexin V^−^/7-AAD^−^), early apoptotic (Annexin V^+^/7-AAD^−^), and late apoptotic/necrotic (Annexin V^+^/7-AAD^+^). Data were analyzed using FlowJo software (Waters Biosciences).

#### *In vivo* oligodendroglioma xenograft model

For SABRAC toxicity study, 16 eight-week-old female SCID mice were randomly divided into four groups having one vehicle control group (n=4) and three SABRAC-treatment groups (n=4 for each dose; 1mg/kg, 5mg/kg and 15mg/kg). Each animal received five intraperitoneally injections per week of SABRAC (SABRAC in 0.5% methylcellulose and 0.2% Tween 80 in PBS) or vehicle control (DMSO in 0.5% methylcellulose and 0.2% Tween 80 in PBS). Routine cage-side observations were made on all animals at least once a day throughout the study for general signs of pharmacologic and toxicologic effects, morbidity, and mortality. Mice were weighed every 3–4 days. 4h after the last injection mice were euthanized and plasma, liver and brain were collected. Brains were processed and analyzed by LC/MS.

For evaluating the efficacy of SABRAC in oligodendroglioma xenograft mice model, 200,000 human TS603 oligodendroglioma cells were intracranially injected in 19 eight-week-old female SCID mice. One week after the injection, mice were randomly divided into two groups having a vehicle control group (n=9) and a SABRAC-treatment group (n=10, 15mg/kg). Each animal received two-three intraperitoneally injections per week of SABRAC (SABRAC in 0.5% methylcellulose and 0.2% Tween 80 in PBS) or vehicle control (DMSO in 0.5% methylcellulose and 0.2% Tween 80 in PBS). Routine cage-side observations and mice weighting were performed as indicated for the toxicity study. Animals were euthanized when reaching the endpoint of deteriorated clinical score and weight-loss (more than 15% of body weight).

### Statistical analysis

Statistical analyses were performed using GraphPad Prism (version 11.0.0). Data are presented as mean ± SD or mean ± SEM as indicated in the corresponding figure legends. The specific statistical tests used for each experiment are described in the figure legends. For comparisons between two groups, unpaired two-tailed Student’s t-tests were used unless otherwise specified. For experiments involving multiple groups, one-way or two-way ANOVA followed by appropriate post hoc multiple-comparison tests was applied as indicated. Lipidomics data were log-transformed prior to statistical analysis where appropriate. RNA-seq differential expression analysis was performed using DESeq2, and pathway analyses (GSEA and IPA) were conducted using their respective statistical frameworks as described in the Methods section. A p value < 0.05 was considered statistically significant.

Dose-response curves and IC_50_ values were calculated using nonlinear regression. A four-parameter logistic model (Concentration-response, variable slope) was fitted by ordinary least squares with strict convergence criteria (maximum 1000 iterations). Viability values were normalized to the mean of untreated controls (0 μM), which was set to 1. The top plateau was constrained to 1 and the bottom plateau constrained to 0 to standardize comparisons across cell lines and to minimize the influence of occasional negative values resulting from background subtraction. No weighting or outlier exclusion was applied, and each replicate was treated as an individual data point. Asymmetrical 95% confidence intervals were calculated. Model adequacy was assessed by inspection of residual plots and parameter stability diagnostics. Alternative fitting strategies, including log10-transformed concentration and 1/Y^2^ weighting, were explored; when these approaches produced unstable parameter estimates, concentration-based regression was retained for consistency across cell lines.

## Supplementary Material

Supplementary Files

This is a list of supplementary files associated with this preprint. Click to download.

• RawdataMuleyetal.2026.xlsx

• OriginalwesternblotsMuleyetal.2026.docx

• SupplementaryfilesMuleyetal.2026.docx

## Data Availability

All datasets generated during this study are available from the corresponding author upon reasonable request.
